# Causal Discovery and Reasoning for Continuous Variables with an Improved Bayesian Network Constructed by Locality Sensitive Hashing and Kernel Density Estimation

**DOI:** 10.3390/e27020123

**Published:** 2025-01-24

**Authors:** Chenghao Wei, Chen Li, Yingying Liu, Song Chen, Zhiqiang Zuo, Pukai Wang, Zhiwei Ye

**Affiliations:** 1 School of Computer Science, Hubei University of Technology, Wuhan 430068, China; chenghao.wei@hbut.edu.cn (C.W.); yingying.liu@hbut.edu.cn (Y.L.); zzq@hbut.edu.cn (Z.Z.); hgcsyzw@hbut.edu.cn (Z.Y.); 2 College of Acupuncture and Moxibustion and Orthopaedics, Hubei University of Chinese Medicine, Wuhan 430065, China; chensong@hbzyydx.wecom.work; 3 Hubei Shizhen Laboratory, Wuhan 430065, China

**Keywords:** Bayesian network, kernel density estimation, locality sensitive hashing, hybrid-based structure learning, mutual information, conditional mutual information, conditional entropy

## Abstract

The structure learning of a Bayesian network (BN) is a crucial process that aims to unravel the complex dependencies relationships among variables using a given dataset. This paper proposes a new BN structure learning method for data with continuous attribute values. As a non-parametric distribution-free method, kernel density estimation (KDE) is applied in the conditional independence (CI) test. The skeleton of the BN is constructed utilizing the test based on mutual information and conditional mutual information, delineating potential relational connections between parents and children without imposing any distributional assumptions. In the searching stage of BN structure learning, the causal relationships between variables are achieved by using the conditional entropy scoring function and hill-climbing strategy. To further enhance the computational efficiency of our method, we incorporate a locality sensitive hashing (LSH) function into the KDE process. The method speeds up the calculations of KDE while maintaining the precision of the estimates, leading to a notable decrease in the time required for computing mutual information, conditional mutual information, and conditional entropy. A BN classifier (BNC) is established by using the computationally efficient BN learning method. Our experiments demonstrated that KDE using LSH has greatly improved the speed compared to traditional KDE without losing fitting accuracy. This achievement underscores the effectiveness of our method in balancing speed and accuracy. By giving the benchmark networks, the network structure learning accuracy with the proposed method is superior to other traditional structure learning methods. The BNC also demonstrates better accuracy with stronger interpretability compared to conventional classifiers on public datasets.

## 1. Introduction

A Bayesian network (BN) is an effective tool for expressing the causal relationships among variables [1]. However, a BN is essentially a statistical modeling tool, and the direction and strength of its edges do not necessarily have causal significance, serving more to describe the correlations between variables. In practical applications, when the research objective is explicitly focused on the causal relationships between variables, causal learning models can be regarded as a specialized form of Bayesian networks, explicitly capturing causal connections between variables within the network structure. As a directed acyclic graph (DAG) based on probability and graph theory, a BN has significant advantages for causal discovery and uncertainty reasoning. Due to their strong representation capability regarding knowledge in terms of data, BNs have been widely applied in industrial monitoring [2], control theory [3], information retrieval, bioinformatics, and computational biology, especially in the field of medical diagnosis and treatment [4].

BN learning is an NP-hard problem. Structure learning can usually be divided into search-based methods, constraint-based methods, and hybrid-based methods [5]. A good search-based method for BN should not only avoid local optima but also consider computational efficiency during structure learning. By using discrete firefly optimization [6], BN structure learning was completed for discrete variables. Combining hill-climbing (HC) with particle swarm optimization (PSO), HC-PSO [7] was proposed for enhancing the searching speed during structure learning. In addition to utilizing the common Bayesian information criterion (BIC) and minimum description length (MDL) metrics, the assessment of the implicit inference framework was employed as the scoring approach to find the most suitable network quickly [8]. These methods have certain issues associated with them. Because of the limitations of the proposed scoring function, it may not fully reflect the true structure of the data. Furthermore, owing to limitations in computational resources or time, search algorithms might not be capable of thoroughly investigating every potential network structure. Constraint-based techniques construct an undirected graph based on the conditional independence relationship between variables and then determine the direction of edges by V-structure recognition [9,10]. Nevertheless, certain edges may remain undetermined due to insufficient statistical evidence or the existence of incomplete or noisy data, potentially impairing performance. Hybrid methods integrate both techniques, utilizing constraint-based strategies to minimize the search area, followed by a search-based technique to identify the best network structure [11]. Recently, hybrid-based methods have led to positive learning achievements and have become a common practice for understanding Bayesian network structures [12]. A novel strategy that combined a dynamic threshold with a triangle-disrupting skeleton learning method was developed to successfully capture the structure of the BN [13]. The scoring and searching functions were applied in the complete node ordering space, and a new type of neighbor operation was proposed to shrink the search space [14].

While many BN structure learning strategies have been tailored for discrete variables, continuous data are more prevalent in many fields, such as medical diagnosis and risk assessment. Learning the structure of Bayesian networks with continuous variables helps improve model accuracy and provides more reliable information for practical decision-making. However, there were only a few studies that investigated the handling of continuous variables. Discretization was usually applied before network structure learning for continuous variables [15]. Not only could it result in the loss of data information, but it might also create misleading dependencies [16]. Many methods have been proposed to compensate for deficiencies. By introducing partial correlation coefficients or mutual information, the constraint-based methods can achieve CI tests for continuous variables to build undirected graph [17]. The d-separation principle enabled the conversion of an undirected graph into a DAG. Based on the kernel generalized variance (KGV), all the discrete and continuous variables were treated on an equal footing as Gaussians in a feature space obtained from Mercer kernels [18]. An efficient method was proposed for selecting the optimal regularization constant on a per node basis by using the L1-regularization path [19]. However, most research works are based on the assumption of a multivariate Gaussian distribution, yet the data in practical applications may not satisfy this assumption. Estimation of Gaussian linear structural equation models could pose serious identifiability problems when the data do not follow a Gaussian distribution [20]. The data may exhibit no Gaussianity. It is difficult to learn the network structure using the traditional index.

KDE is a typical non-parametric estimation method. It is widely used in various learning tasks [21]. It does not rely on assumptions regarding the sample distribution. An improved constraint-based BN learning method using the Gaussian kernel probability density estimator has been proposed for handling continuous variables [22]. It employs KDE to compute mutual information. The correctness of the final BN structure is closely tied to the results of CI testing. In real-world scenarios, the outcomes of CI testing might not be entirely dependable due to several influences, including the quality of the data, the size of the sample, and the nature of the data distribution. Certain edge orientations in a BN could be difficult to identify. The KDE computational demands are a factor requiring consideration. Building a BN for continuous variables effectively remains a significant problem. For tackling these issues, our contributions can be summarized as follows:

- This paper offers new mutual information and conditional mutual information based on KDE for CI testing. The mathematical formula is derived based on the Gaussian kernel. By using such information formulas, a new conditional entropy is calculated, which can be used as a scoring function. The index is used for evaluating the uncertainty degree of a node by providing a set of parent nodes. The index can be used as an effective tool for deciding the parent nodes of a given node. This method can more accurately handle continuous variables without making assumptions about the data distribution, avoiding information loss and erroneous dependencies caused by discretization, thereby improving the accuracy of network structure learning.
- This paper lowers the computational complexity of KDE. The new KDE introduces LSH functions to accelerate the computational speed of the Gaussian KDE. Without sacrificing estimation accuracy, it reduces the computational cost from $O(n^{2})$ to O(nL) where *n* is the number of samples and *L* is the number of hashing functions. This improvement significantly enhances computational efficiency, providing a more practical and operable method for datasets in real-world applications.
- By treating the class attribute as parents of all non-class attributes, this paper provides a new method for BNC, which considers the dependency of variables. In the application, our BN classification model has a higher performance in classifying actual data compared to classic classifiers. Due to the graph structure, it can effectively describe the correlation between attributes, which greatly improves the interpretability of the model. The advantage improves its usability in application scenarios, particularly for the medical disease data.

This paper consists of the following parts. In Section 2, we provide a detailed discussion of the relevant research works. In Section 3, we discuss the basic Bayesian network learning methods. In Section 4, we offer an in-depth description of the hybrid BN structure learning technique using the LSH-based KDE (LSHKDE). In Section 5, we present the principles for building a BNC. In Section 6, we carry out a range of experimental works. The final section entails the conclusion.

## 2. Related Work

### 2.1. BN Structure Learning Methods for Discrete Variables

Constraint-based methods rely heavily on CI tests to determine the relationships between variables. The Incremental Association Markov Blanket (IAMB) algorithm [23], which was proposed by Tsamardinos et al., is a notable example that aims to learn a Markov blanket for each variable in the network. The IAMB algorithm iteratively adds variables to the Markov blanket of a target variable based on their strength of association with the target variable, while ensuring that the added variables are conditionally independent of the target variable given the current Markov blanket. However, one limitation of this algorithm is that the CI tests are performed given the entire Markov blanket, which can significantly increase the number of conditional independence test that needs to be performed. The Hiton-PC algorithm [24], proposed by Aliferis et al., aims to address this issue by separating the steps of adding parent–child nodes and eliminating non-parent nodes. In the algorithm, the conditional test set is always the empty set, making the algorithm easier to implement. However, like IAMB, Hiton-PC still relies on CI tests to determine the relationships between variables. To address the selection biases introduced by complex data, Marella and Vicard proposed an improved algorithm for PC [25], which addresses the selection biases introduced by complex data by using modified independence tests based on resampling techniques.

Search-based methods for BN structure learning are typically divided into two categories, which are model selection and model optimization. Model selection methods aim to construct a scoring function that evaluates the goodness of fit of a given BN structure to the data, and then use this function to search for the optimal BN structure. The mentioned BIC, MDL and the Akaike Information Criterion (AIC) are used as scoring criteria for model selection. These criteria are primarily designed for discrete variables. Heckerman et al. proposed the HC algorithm [1], which learns the structure of Bayesian networks by adding, removing, and inverting edges and it is easy to achieve a local optimal solution. Cooper and Herskovits proposed the K2 search algorithm [26]. This algorithm starts from a network node. Based on a predetermined node order and the maximum number of parent nodes, it selects the highest-scoring nodes as parents. However, finding the optimal node order is often an NP-hard problem, which can significantly impact the accuracy of the network structure. Behiati and Beigy proposed an improved K2 algorithm [27]. The algorithm first constructs a graph from the data, then extracts the strongly connected components (SCCs) of the graph. The extracted SCCs are used to generate an initial ordering of the nodes, which is then provided to the K2 algorithm to learn the structure of the BN. This approach can help to reduce the impact of the node ordering on the performance of the K2 algorithm and can improve the accuracy of the learned BN structure.

Singh and Valtorta proposed the first hybrid algorithm [28] that used the improved PC algorithm [29] to obtain the node order and then used the K2 algorithm to learn the optimal network structure. However, the algorithm has shortcomings in computational efficiency and model optimization. Tsamardinos et.al proposed the classical max–min hill-climbing (MMHC) algorithm [30], which used the max–min parents and children (MMPC) algorithm [31] to obtain the set of candidate parent–child nodes and applied the greedy HC algorithm for searching the optimal network structure. In terms of the quality of the constructed network, the MMHC algorithm outperforms many other algorithms. Song et al. used MMHC to construct a BN to explore the network relationship between multimorbidity and its correlates [32], demonstrating superiority over traditional logistic regression models. Additionally, Bayesian inference was employed to perform risk reasoning for multimorbidity, which aligns better with clinical practice and shows promising application prospects.

### 2.2. BN Structure Learning Methods for Continuous Variables

For continuous variables, a handling is to discretize continuous variables. Chen et al. introduced a principled Bayesian discretization method [33]. It deals with continuous variables in a BN with quadratic complexity instead of the cubic complexity of other standard techniques. Another method avoids discretization and directly handles continuous variables.

In search-based methods, different CI metrics were designed for the structural learning of continuous variables. The partial correlation coefficient can be used for measuring the correlation between nodes. Wang and Chan proposed a heuristic partial correlation-based (HP) algorithm [34], which was based on a three-stage framework. It was finalized by edge orienting according to the complex orientation rules. However, it had a high time complexity. Wang and Chan introduced a two-stage algorithm [35]. This algorithm initially constructed an undirected graph based on partial correlation coefficients. It then removed redundant edges using partial correlation-based CI tests. Finally, it identified V-structures and oriented the output BN. The algorithm still has a high time complexity. Mutual information can measure the correlations between nodes naturally. Huegle proposed a KNN-based conditional mutual information estimator as an indicator for CI tests to learn a BN structure [36]. It employed a KNN-based local permutation scheme to calculate *p*-values, suitable for mixed-value data. Entropy under non-parametric estimation conditions could also be used to calculate mutual information. Dávid et al. proposed a simple and computationally efficient non-parametric estimation of entropy and mutual information [37]. The estimator was calculated as the sum of the p-th powers of the Euclidean lengths of edges in the generalized nearest neighbor graph and the empirical joint of the samples. Nguyen et al. compared the effectiveness of mutual information calculated by counting methods and KDE methods in feature selection using particle swarm optimization as the search mechanism [38]. The results indicated that KDE worked well on both continuous and discrete datasets. Charikar and Siminelakis studied the problem of designing a data structure that given a dataset and a kernel function, returned approximations to the kernel density of a query point in sublinear time [39]. They introduced a class of unbiased estimators for kernel density implemented through LSH and give general theorems bounding the variance of such estimators. Jiang et al. proposed a method using the Gaussian KDE to calculate entropy values, which in turn are used to compute conditional mutual information [22]. It measured the correlation between different variables, thereby improving the learning accuracy of constraint-based BN structure learning methods.

In search-based methods, the scoring function was modified to accommodate structure learning of continuous variables. Geiger and Heckerman studied the problem of structural learning for Gaussian networks [40]. Assuming that continuous data were sampled from multivariate Gaussian distributions, a scoring metric was presented for continuous variables. They also a holistic scoring search method based on probabilistic models. However, the scoring function is too complex to be practical for solving real-world problems. Andrews et al. proposed the Conditional Gaussian (CG) scoring method and the Mixed Variable Polynomial score for learning BN networks with continuous and discrete variables [41]. The scoring method offered an efficient option. The second scoring method allowed for a broader range of modeling relationships but was slower than CG.

In hybrid-based methods, the CI test metrics and scoring functions were modified to make them suitable for continuous variable. Schmidt et al. proposed the L1MB algorithm [19], which could handle continuous data following a multivariate Gaussian distribution generated by linear structural equation models. This algorithm used the least angle regression to search for candidate neighbors of each node, identifying the most suitable set of nodes as its candidate neighbors. Then, within this constrained space, it employed an HC search algorithm to obtain the final network structure. However, when the number of network nodes was large, the learning accuracy of the network was not high. Yang et al. proposed a new algorithm for BN structure learning, named partial correlation-based (PCB) [42]. This algorithm effectively combined local learning and partial correlation techniques. It reconstructed the BN skeleton based on the principles of partial correlation. Then, it performed a greedy HC search to determine the directions of the network edges. But, if the inverse of the correlation coefficient matrix *R* does not exist, the algorithm will fail.

## 3. Bayesian Network Learning

We first provide a general definition of BN and causal model, and then we analyze the principles of a hybrid BN structure learning algorithm.

### 3.1. Bayesian Network

BN, also known as belief network, is a probability graph model that describes the dependency relationships between random variables. The mathematical representation of BN is given in the following definition.

**Definition** **1.** *A BN is a binary set BN=(G,θ), where G=(X,E) is a DAG, $\theta =\{\theta^{1},\theta^{2},\ldots ,\theta^{n}\}$ is the Conditional Probability Distribution (CPD), the random variables is $X=\{x_{1},x_{2},\ldots ,x_{n}\}$ in the graph. The CPD of each variable has the following form:*

(1) $$\theta^{i}=P_{x_{i}}=P\left(x_{i}|Pa\left(x_{i}\right)\right)$$

Usually, BN=(G,θ) and $BN=(G,\left\{P_{x_{i}},\ldots ,P_{x_{n}}\right\})$ are equivalent. A BN satisfies the Markov property by giving the parent node set of any variable, $\forall x_{i}\in X$; the non-descendant nodes are represented by $ND(x_{i})$ and they satisfy the condition $x_{i}⊥ND\left(x_{i}\right)|Pa\left(x_{i}\right)$. According to the Markov property, the joint probability distribution of BN can be simplified as the following equation [43]:

(2) $$P\left(X\right)=P\left(x_{1},\ldots ,x_{n}\right)=\prod_{i=1}^{n}P(x_{i}|Pa\left(x_{i}\right))$$

where $Pa(x_{i})$ represents the parent node set of node $x_{i}$ in *G*. The following definition offers a clear description for a causal model [44,45].

**Definition** **2.** *A causal model is a tuple M = (G, θ), where θ is a set of parameters compatible with G, used to quantitatively describe the dependencies among variables. Specifically, for each variable $x_{i}$, the relationship between $x_{i}$ and its parent nodes $Pa(x_{i})$ is described by a function $x_{i}=f_{x_{i}}\left(Pa\left(x_{i}\right),u_{x_{i}}\right)$ where $Pa(x_{i})$ represents the values of its parent nodes, and $u_{x_{i}}$ is a random disturbance term that follows a specified probability distribution $P(u_{x_{i}})$.*

### 3.2. Hybrid BN Structure Learning Algorithm

The MMHC algorithm is a typical hybrid BN structure algorithm, which includes a constraint phase and a search phase. In the constraint phase, the goal is to determine the parent and child nodes for each node, forming the skeleton of an undirected graph in the *G*. In the search phase, the HC algorithm is used to do search job within the obtained skeleton from the constraint phase. This search determines the directions of the edges to achieve the optimal graph structure $G_{opt}$, thereby significantly improving the speed of network construction.

The MMPC algorithm adopts a max–min strategy in the growing phase, heuristically introducing nodes into the candidate parent–child (CPC) set of the target node $x_{t}$. Specifically, assuming that $CS=\{cs_{1},cs_{2},\ldots ,cs_{q}\}$, CS⊆CPC as the conditional set, MMPC calculates the correlation between each node $x_{i}$ and the target node $x_{t}$, and selects the maximum node in the set with the lowest correlation to the CPC. If the nodes in the CPC are no longer changed, the growing phase will be terminated. In the shrinking phase, nodes that should not be selected during the growing phase are removed carefully. If there is a node $x_{i}$ in the CPC of the target node $x_{t}$, the CI test between $x_{i}$ and $x_{t}$ is conducted using the independence function $Ind(x_{i},x_{t}|CS)$. The CS is the condition set. If they are independent, the node $x_{i}$ will be removed from the CPC. During the search phase of MMHC, the scoring function, as an objective function, is solved by using the HC strategy. Subsequently, the edge directions of the undirected graph are manipulated by adding, deleting, and reversing them to achieve the optimal network structure.

## 4. Hybrid BN Structure Learning Based on LSHKDE

This paper uses a method based on Gaussian KDE to calculate mutual information, conditional mutual information, and conditional entropy. It replaces the chi-square test used in the constraint phase of the MMHC algorithm and the scoring function used in the search phase.

### 4.1. Gaussian KDE

Unlike parametric statistical methods, KDE does not depend on prior assumptions or prior knowledge [21]. It can obtain the characteristics of the density distribution of the data samples themselves. It does not require assumptions about the data distribution model, making this method suitable for various types of data distributions. Assuming $S=\left\{S_{1},\ldots ,S_{i},\ldots ,S_{n}\right\}$, $S_{i}\in R^{d}$, each variable independently and identically obeys an unknown multivariate probability density function *F*. The expression for multivariate standard KDE is shown below [46]:

(3) $$P\left(\vec{x}\right)=\frac{1}{nh^{d}}\sum_{i=1}^{n}\prod_{j=1}^{d}K(\frac{x_{j}-S_{ij}}{h_{j}})$$

where $P(\vec{x})$ is the probability density function obtained from KDE, K(u) is the kernel function, *h* is the window width or bandwidth, and *n* is the sample size. The kernel function of KDE has multiple option; the most common one is the Gaussian kernel [47].

(4) $$K\left(u\right)=\frac{1}{\sqrt{2\pi }}e^{-\frac{u^{2}}{2}}$$

By applying Equations (3) and (4), the KDE with the multivariate Gaussian kernel is shown in the following equation:

(5) $$P\left(\vec{x}\right)=\frac{1}{n{\left(h\sqrt{2\pi }\right)}^{d}}\sum_{i=1}^{n}\prod_{j=1}^{d}e^{-\frac{{\left(x_{j}-S_{ij}\right)}^{2}}{2h^{2}}}$$

Relevant dependency indicators can be further calculated using the estimated density distribution.

### 4.2. Mutual Information and Conditional Mutual Information Based on KDE

Differential entropy is a measure of the uncertainty for continuous random variables [48].

(6) $$H\left(x_{1}\right)=-\int P\left(x_{1}\right)\log P\left(x_{1}\right)dx_{1}$$

Joint differential entropy is used to evaluate the uncertainty between multiple continuous random variables [49]. It is a measure of the information entropy of the joint probability distribution for the multiple random variables. For two given random variables $x_{1}$ and $x_{2}$, their joint probability distribution is $P(x_{1},x_{2})$, and then the joint differential entropy $H(x_{1},x_{2})$ is defined by the following equation:

(7) $$H\left(x_{1},x_{2}\right)=-\int \;\;\;\int P\left(x_{1},x_{2}\right)\log P\left(x_{1},x_{2}\right)dx_{1}dx_{2}$$

Mutual information and conditional mutual information can be obtained from Equations (6) and (7), as shown in the following equations [22]:

(8) $$I(x_{1},x_{2})=H\left(x_{1}\right)+H\left(x_{2}\right)-H\left(x_{1},x_{2}\right)$$

(9) $$I\left(x_{1},x_{2}|x_{3}\right)=H\left(x_{1},x_{3}\right)+H\left(x_{2},x_{3}\right)-H\left(x_{3}\right)-H\left(x_{1},x_{2},x_{3}\right)$$

The simplification of the above equations results in the following equations [50]:

(10) $$I\left(x_{1},x_{2}\right)=\int \;\;\;\int P\left(x_{1},x_{2}\right)\log\frac{P(x_{1},x_{2})}{P\left(x_{1}\right)P\left(x_{2}\right)}dx_{1}dx_{2}$$

(11) $$I\left(x_{1},x_{2}|x_{3}\right)=\int \;\;\;\int \;\;\;\int P\left(x_{1},x_{2},x_{3}\right)\log\frac{P(x_{1},x_{2}|x_{3})}{P\left(x_{1}|x_{3}\right)P\left(x_{2}|x_{3}\right)}dx_{1}dx_{2}dx_{3}$$

where $P(x_{1})$, $P(x_{1},x_{2})$, and $P(x_{1},x_{2},x_{3})$ can be directly calculated using Equation (5). The mutual information and conditional mutual information are shown in the following equations:

(12) $$I\left(x_{1},x_{2}\right)=\frac{1}{n}\sum_{i=1}^{n}\log\frac{n\sum_{j=1}^{n}e^{-\frac{{\left(x_{i1}-S_{j1}\right)}^{2}+{\left(x_{i2}-S_{j2}\right)}^{2}}{2h^{2}}}}{\sum_{j=1}^{n}e^{-\frac{{\left(x_{i1}-S_{j1}\right)}^{2}}{2h^{2}}}\sum_{j=1}^{n}e^{-\frac{{\left(x_{i2}-S_{j2}\right)}^{2}}{2h^{2}}}}$$

(13) $$I\left(x_{1},x_{2}|x_{3}\right)=\frac{1}{n}\sum_{i=1}^{n}\log\frac{\sum_{j=1}^{n}e^{-\frac{{\left(x_{i1}-S_{j1}\right)}^{2}+{\left(x_{i2}-S_{j2}\right)}^{2}+{\left(x_{i3}-S_{j3}\right)}^{2}}{2h^{2}}}\sum_{j=1}^{n}e^{-\frac{{\left(x_{i3}-S_{j3}\right)}^{2}}{2h^{2}}}}{\sum_{j=1}^{n}e^{-\frac{{\left(x_{i1}-S_{j1}\right)}^{2}+{\left(x_{i3}-S_{j3}\right)}^{2}}{2h^{2}}}\sum_{j=1}^{n}e^{-\frac{{\left(x_{i2}-S_{j2}\right)}^{2}+{\left(x_{i3}-S_{j3}\right)}^{2}}{2h^{2}}}}$$

By using Equations (12) and (13), we can deduce that the time complexity of both mutual information and conditional mutual information is $O(n^{2})$.

### 4.3. Conditional Entropy Based on KDE

Conditional entropy can be considered as the uncertainty degree of node *X* given a set of parent nodes. Naturally, conditional entropy h(X|Π) can be used as a scoring function for selecting a set of parent nodes for each node in the network. Conditional entropy is defined in the following equation [51].

(14) $$h_{score}\left(x_{i}|\Pi_{i}\right)=H\left(x_{i},\Pi_{i}\right)-H\left(\Pi_{i}\right)$$

where $H(x_{i},\Pi_{i})$ and $H(\Pi_{i})$ are calculated using Equations (5)–(7). Here, the parent node set of node *X* is denoted as Π. It is obtained from the corresponding CPC set. When Π contains zero nodes, the CPC set may not be empty.

During the search phase, if node $x_{1}$ has no parent node, i.e., Π=∅, the scoring function is the differential entropy of this node $x_{1}$, which can be obtained by using Equations (5) and (6).

(15) $$h_{score}\left(x_{1}|\Pi \right)=H\left(x_{1}\right)=-\frac{1}{n}\sum_{i=1}^{n}\log\frac{\sum_{j=1}^{n}e^{-\frac{{\left(x_{i1}-S_{j1}\right)}^{2}}{2h^{2}}}}{nh\sqrt{2\pi }}$$

Suppose $CPC_{x_{1}}=\left\{x_{2},x_{3}\right\}$ that indicates the CPC set of $x_{1}$, if $x_{2}$ is the parent node of $x_{1}$, i.e., $\Pi_{1}=\left\{x_{2}\right\}$, the scoring function can be obtained using Equations (5)–(7).

(16) $$h_{score}\left(x_{1}|\Pi_{1}\right)=H\left(x_{1},x_{2}\right)-H\left(x_{2}\right)=\frac{1}{n}\sum_{i=1}^{n}\log\frac{h\sqrt{2\pi }\sum_{j=1}^{n}e^{-\frac{{\left(x_{i2}-S_{j2}\right)}^{2}}{2h^{2}}}}{\sum_{j=1}^{n}e^{-\frac{{\left(x_{i1}-S_{j1}\right)}^{2}+{\left(x_{i2}-S_{j2}\right)}^{2}}{2h^{2}}}}$$

If $h_{score}\left(x_{1}|\Pi_{1}\right)<h_{score}\left(x_{1}|\Pi \right)$ is satisfied, then $x_{2}$ will be the parent node of $x_{1}$. For a new set of parent nodes ${\Pi_{1}}^{\prime }=\left\{x_{2},x_{3}\right\}$, the scoring function can be calculated using a similar way.

(17) $$h_{score}\left(x_{1}|{\Pi_{1}}^{\prime }\right)=H\left(x_{1},x_{2},x_{3}\right)-H\left(x_{2},x_{3}\right)=\frac{1}{n}\sum_{i=1}^{n}\log\frac{h\sqrt{2\pi }\sum_{j=1}^{n}e^{-\frac{{\left(x_{i2}-S_{j2}\right)}^{2}+{\left(x_{i3}-S_{j3}\right)}^{2}}{2h^{2}}}}{\sum_{j=1}^{n}e^{-\frac{{\left(x_{i1}-S_{j1}\right)}^{2}+{\left(x_{i2}-S_{j2}\right)}^{2}+{\left(x_{i3}-S_{j3}\right)}^{2}}{2h^{2}}}}$$

If the $h_{score}\left(x_{1}|{\Pi_{1}}^{\prime }\right)<h_{score}\left(x_{1}|\Pi_{1}\right)$ is satisfied, it indicates $x_{3}$ will be considered as one of the parent nodes of $x_{1}$. It needs to be added to the parent node set $\Pi_{1}$. Otherwise, if the newly added node $x_{3}$ cannot reduce the uncertainty of node $x_{1}$, it should be discarded. According to the conditional entropy, there is a strong direct causal relationship between node $x_{3}$ and node $x_{1}$. Therefore, this scoring method can directly quantify the degree of uncertainty and obtain causal relationships.

Generally there are *K* nodes in ${\Pi_{1}}^{\prime }$, $H(x_{1},{\Pi_{1}}^{\prime })$ will be a k+1 joint differential entropy. It needs K+1 joint Gaussian KDE for the evaluation. By using Equations (5)–(7) for calculation, the time complexity of conditional entropy is $O(n^{2})$. The time complexity of the MMHC-KDE algorithm is $O(m2^{\left|CPC\right|}n^{2})$, where |CPC| represents the number of parent–child nodes, *m* is the number of variables, *n* is the number of samples.

### 4.4. Gaussian KDE Based on LSH

For a high-dimensional dataset with large samples, Gaussian KDE typically exhibits poor scalability in terms of computation and memory. To address the problem by employing LSH, an accelerated KDE will be acquired. It significantly reduces the computation time and memory space while maintaining estimation accuracy. The new method is suitable for large-scale data processing. The approximate definition of KDE is provided as follows.

**Definition** **3.** *Assume $S=\left\{S_{1},\ldots ,S_{i},\ldots ,S_{n}\right\}$, $S_{i}\in R^{d}$ is a sample. By using two parameters ε,τ∈(0,1), we can construct a data structure that ensures each query point $\vec{x}\in R^{d}$ satisfies $KDE(\vec{x})⩾\tau$, and the approximated estimation $\bar{KDE}\left(\vec{x}\right)$ can be obtained under a given probability [39].*

(18) $$\left(1-\varepsilon \right)KDE\left(\vec{x}\right)⩽\bar{KDE}\left(\vec{x}\right)⩽\left(1+\varepsilon \right)KDE\left(\vec{x}\right)$$

The traditional calculation of $KDE(\vec{x})$ performs well with all samples. According to the standard concentration inequalities, the above approximation can be achieved by uniformly and randomly selecting $O(\frac{1}{\tau }\frac{1}{\varepsilon^{2}})$ points from *S* to evaluate the estimation observer $\vec{x}$. It returns the average as an approximate calculation. A hash mapping $hash\left(\vec{x}\right):\vec{x}\to \left\{1,\ldots ,R\right\}\in Z$ is a function that maps the input $\vec{x}$ to an integer in the range [1,R]. An LSH family is a set of hash functions, which are defined as follows.

**Definition** **4.** *For a sensitive hash ethnic group $\left(R1,cR1,k_{1},k_{2}\right)$ with a given distance function $d\left(\cdot ,\cdot \right)$, if hash∈HASH satisfies the following properties with any two points x and S, the HASH is called a $\left(R1,cR1,k_{1},k_{2}\right)$ sensitive hash family [52].*

**Figure 1:**
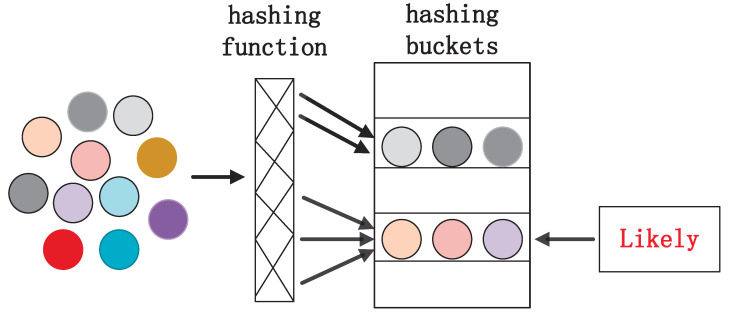
Locality sensitive hashing schematic.

- If d(x,S)⩽R1, and then $\underset{hash∼HASH}{\mathrm{Pr}}\left[hash\left(x\right)=hash\left(S\right)\right]\geq k_{1}$
- If d(x,S)⩾cR1, and then $\underset{hash∼HASH}{\mathrm{Pr}}\left[hash(x)=hash(S)\right]\leq k_{2}$

where $\underset{hash∼HASH}{\mathrm{Pr}}\left[hash\left(x\right)=hash\left(S\right)\right]$ represents the probability that *x* and *S* will collide. By choosing a suitable hash function, similar data objects are hashed into the same hash bucket with a higher probability as shown in Figure 1. For a Gaussian KDE, it is expressed as $K\left(x,S\right)=e^{-||x-S|{|}^{2}/h^{2}}$ using the Euclidean distance. Its optimal LSH family can be obtained using the spherical distance. The collision probability $\underset{hash∼HASH}{Pr}\left[hash\left(x\right)=hash\left(S\right)\right]$ will not increase if the distance $||x-{S||}_{2}$ is satisfied.

**Theorem** **1.** *For any R2>0, there exists a hash set such that for any $x,S\in R^{d}$, when ${∥x-S∥}_{2}\leq R2$ holds, then the following bound holds [53].*

(19) $$e^{-||x-{S||}_{2}^{2}}\cdot e^{-o(R^{\frac{4}{3}}\log R2)}⩽\underset{hash∼HASH}{\mathrm{Pr}}\left[hash\left(x\right)=hash\left(S\right)\right]⩽e^{-||x-{S||}_{2}^{2}}\cdot e^{o(R^{\frac{4}{3}}\log R2)}$$

This paper applies the LSH function to calculate Gaussian KDE. The calculation is primarily divided into preprocessing and query. In the preprocessing phase, *L* independently selected hash functions HASH are used to map the data sample set *S* to the corresponding hash bucket. This process aims to construct an efficient data index for future query operations. The purpose of the query phase is to accurately locate the given query points in the hash structure, thereby achieving fast and effective KDE. Algorithm 1 provides a detailed description of the calculation process.

In the preprocessing phase, $L=O(n/\left(\sqrt{\tau }\varepsilon^{2}\right))$ hash functions $hash_{1},\ldots ,hash_{L}$ is sampled from the HASH set. The $hash_{j}\left(S_{i}\right)$ function is the *j*th hash mapping for the *i*th sample. The hash value of each data point is stored with a probability of $\delta =1/(n\sqrt{\tau })$ [53]. Therefore, the preprocessing time is $O(T_{H}/\left(\tau \cdot \varepsilon^{2}\right))$, and the spatial utilization is $O(S_{H}/\left(\tau \cdot \varepsilon^{2}\right))$. The $T_{H}$ is the time required to calculate the hash value of a point, and $S_{H}$ represents the space required to store that point.
**Algorithm 1**: LSHKDE algorithm**Input:** Dataset $S=\left\{S_{1},\ldots ,S_{i},\ldots ,S_{n}\right\}$, $S_{i}\in R^{d}$; Query data $x\in R^{d}$; Kernel $K\left(\cdot ,\cdot \right)$; LSH family HASH; Integer 1≤L≤n; Bandwidth *h*. **Output:** The estimation probability density P(x) 1: **Preprocessing phase:** 2: Initialize *L* hash functions $HASH=[hash_{1},hash_{2},\ldots ,hash_{L}]$ 3: **for** i 1 to n **do** 4:     **for** j 1 to L **do** 5:         Randomly hash $S_{i}$ according to the hash function $hash_{j}$ from HASH and save it to $hash_{j}\left(S_{i}\right)$ 6:     **end for** 7: **end for** 8: **Query phase:** 9: **for** k 1 to L **do** 10:     Sample a uniformly random point $S^{\prime }$ from the bin set $bin_{k}\left(x\right)=\left\{S|hash_{k}\left(x\right)=hash_{k}\left(S\right)\right\}$ 11:     $Z_{k}\to \frac{|bin_{k}\left(x\right)|K\left(x,S^{\prime }\right)}{L\mathrm{Pr}[hash(x)=hash(S)]}$ and calculate the Pr[hash(x)=hash(S)] by using Equation (19) 12: **end for** 13: Return $P\left(x\right)\to \frac{1}{{\left(\sqrt{2\pi }h\right)}^{d}L}\sum_{k=1}^{L}Z_{k}$


In the query phase, for a given query point *x*, define a set of samples $bin_{k}\left(x\right)=\left\{S|hash_{k}\left(S\right)=hash_{k}\left(x\right)\right\}$, the $hash_{k}\left(S\right)$ is the hash value of the samples *S* by using the hash function $hash_{k}$. The $hash_{k}\left(x\right)$ is the hash value calculated for a query point *x*. The set $bin_{k}\left(x\right)$ is a collection of all data points whose hash values through $hash_{k}$ are the same as those of *x*. Randomly select a data point $S^{\prime }$ from the set $bin_{k}\left(x\right)$ and calculate $Z_{k}=\frac{|bin_{k}\left(x\right)|K\left(x,S^{\prime }\right)}{L\mathrm{Pr}[hash(x)=hash(S)]}$, where $|bin_{k}\left(x\right)|$ represents the number of elements in the set $bin_{k}\left(x\right)$. If $bin_{k}\left(x\right)$ is empty, then $Z_{k}=0$. The final KDE estimate is $P\left(x\right)=\frac{1}{{\left(\sqrt{2\pi }h\right)}^{d}L}\sum_{k=1}^{L}Z_{k}$, with a query time of O($\left(T_{H}+T_{M}\right)/\left(\sqrt{\tau }\varepsilon^{2}\right)$), where $T_{M}$ is the time cost of the $K(x,S^{\prime })$ for a single pair.

### 4.5. Mutual Information and Conditional Entropy Based on LSHKDE

In the MMHC algorithm, by using Gaussian LSHKDE, mutual information, conditional mutual information, and conditional entropy are calculated. The new calculation is shown in the below equations.

(20) $$P\left(x_{1}\right)=\frac{1}{L^{2}\sqrt{2\pi }h}\sum_{i=1}^{L}\frac{|bin_{i}\left(x_{1}\right)|e^{-\frac{{\left(x_{1}-S_{i1}\right)}^{2}}{2h^{2}}}}{\mathrm{Pr}[hash\left(x_{1}\right)=hash\left(S_{i1}\right)]}$$

(21) $$P\left(x_{1},x_{2}\right)=\frac{1}{L^{2}{\left(\sqrt{2\pi }h\right)}^{2}}\sum_{i=1}^{L}\frac{|bin_{i}\left(x_{1},x_{2}\right)|e^{-\frac{{\left(x_{1}-S_{i1}\right)}^{2}+{\left(x_{2}-S_{i2}\right)}^{2}}{2h^{2}}}}{\mathrm{Pr}[hash\left(x_{1},x_{2}\right)=hash\left(S_{i1},S_{i2}\right)]}$$

By using Equations (20) and (21), we can obtain the corresponding mutual information and conditional mutual information as shown in the following equations.

(22) $$I\left(x_{1},x_{2}\right)=\frac{1}{n}\sum_{i=1}^{n}\log\frac{L^{2}\sum_{j=1}^{L}\frac{|bin_{j}\left(x_{1},x_{2}\right)|e^{-\frac{{\left(x_{i1}-S_{j1}\right)}^{2}+{\left(x_{i2}-S_{j2}\right)}^{2}}{2h^{2}}}}{\mathrm{Pr}[hash\left(x_{i1},x_{i2}\right)=hash\left(S_{j1},S_{j2}\right)]}}{\sum_{j=1}^{L}\frac{|bin_{j}\left(x_{1}\right)|e^{-\frac{{\left(x_{i1}-S_{j1}\right)}^{2}}{2h^{2}}}}{\mathrm{Pr}[hash\left(x_{i1}\right)=hash\left(S_{j1}\right)]}\sum_{j=1}^{L}\frac{|bin_{j}\left(x_{2}\right)|e^{-\frac{{\left(x_{i2}-S_{j2}\right)}^{2}}{2h^{2}}}}{\mathrm{Pr}[hash\left(x_{i2}\right)=hash\left(S_{j2}\right)]}}$$

(23) $$I\left(x_{1},x_{2}|x_{3}\right)=\frac{1}{n}\sum_{i=1}^{n}\log\frac{\sum_{j=1}^{L}\frac{|bin_{j}\left(x_{1},x_{2},x_{3}\right)|e^{-\frac{{\left(x_{i1}-S_{j1}\right)}^{2}+{\left(x_{i2}-S_{j2}\right)}^{2}+{\left(x_{i3}-S_{j3}\right)}^{2}}{2h^{2}}}}{\mathrm{Pr}[hash\left(x_{i1},x_{i2},x_{i3}\right)=hash\left(S_{j1},S_{j2},S_{j3}\right)]}\sum_{j=1}^{L}\frac{|bin_{j}\left(x_{3}\right)|e^{-\frac{{\left(x_{i3}-S_{j3}\right)}^{2}}{2h^{2}}}}{\mathrm{Pr}[hash\left(x_{i3}\right)=hash\left(S_{j3}\right)]}}{\sum_{j=1}^{L}\frac{|bin_{j}\left(x_{1},x_{3}\right)|e^{-\frac{{\left(x_{i1}-S_{j1}\right)}^{2}+{\left(x_{i3}-S_{j3}\right)}^{2}}{2h^{2}}}}{\mathrm{Pr}[hash\left(x_{i1},x_{i3}\right)=hash\left(S_{j1},S_{j3}\right)]}\sum_{j=1}^{L}\frac{|bin_{j}\left(x_{2},x_{3}\right)|e^{-\frac{{\left(x_{i2}-S_{j2}\right)}^{2}+{\left(x_{i3}-S_{j3}\right)}^{2}}{2h^{2}}}}{\mathrm{Pr}[hash\left(x_{i2},x_{i3}\right)=hash\left(S_{j2},S_{j3}\right)]}}$$

The new conditional entropy equations can be derived from Equations (20) and (21). The corresponding scoring functions are shown in the following equations.

(24) $$h_{score}\left(x_{1}|\Pi \right)=H\left(x_{1}\right)=-\frac{1}{n}\sum_{i=1}^{n}\log\frac{\sum_{j=1}^{L}\frac{|bin_{j}\left(x_{1}\right)|e^{-\frac{{\left(x_{i1}-S_{j1}\right)}^{2}}{2h^{2}}}}{\mathrm{Pr}[hash\left(x_{i1}\right)=hash\left(S_{j1}\right)]}}{L^{2}h\sqrt{2\pi }}$$

(25) $$h_{score}\left(x_{1}|\Pi_{1}\right)=H\left(x_{1},x_{2}\right)-H\left(x_{2}\right)=\frac{1}{n}\sum_{i=1}^{n}\log\frac{\sqrt{2\pi }h\sum_{j=1}^{L}\frac{|bin_{j}\left(x_{2}\right)|e^{-\frac{{\left(x_{i2}-S_{j2}\right)}^{2}}{2h^{2}}}}{\mathrm{Pr}[hash\left(x_{i2}\right)=hash\left(S_{j2}\right)]}}{\sum_{j=1}^{L}\frac{|bin_{j}\left(x_{1},x_{2}\right)|e^{-\frac{{\left(x_{i1}-S_{j1}\right)}^{2}+{\left(x_{i2}-S_{j2}\right)}^{2}}{2h^{2}}}}{\mathrm{Pr}[hash\left(x_{i1},x_{i2}\right)=hash\left(S_{j1},S_{j2}\right)]}}$$

(26) $$h_{score}\left(x_{1}|\Pi_{1}^{\prime }\right)=H\left(x_{1},x_{2},x_{3}\right)-H\left(x_{2},x_{3}\right)=\frac{1}{n}\sum_{i=1}^{n}\log\frac{\sqrt{2\pi }h\sum_{j=1}^{L}\frac{|bin_{j}\left(x_{2},x_{3}\right)|e^{-\frac{{\left(x_{i2}-S_{j2}\right)}^{2}+{\left(x_{i3}-S_{j3}\right)}^{2}}{2h^{2}}}}{\mathrm{Pr}[hash\left(x_{i2},x_{i3}\right)=hash\left(S_{j2},S_{j3}\right)]}}{\sum_{j=1}^{L}\frac{|bin_{j}\left(x_{1},x_{2},x_{3}\right)|e^{-\frac{{\left(x_{i1}-S_{j1}\right)}^{2}+{\left(x_{i2}-S_{j2}\right)}^{2}+{\left(x_{i3}-S_{j3}\right)}^{2}}{2h^{2}}}}{\mathrm{Pr}[hash\left(x_{i1},x_{i2},x_{i3}\right)=hash\left(S_{j1},S_{j2},S_{j3}\right)]}}$$

By the analysis of the above equations, the time complexity of mutual information, conditional mutual information, and conditional entropy are all O(nL). Compared to the original KDE, it is significantly improved. The time complexity of the entire algorithm MMHC-LSHKDE is $O(m2^{\left|CPC\right|}nL)$, where |CPC| represents the number of parent–child nodes, and m is the number of variables.

### 4.6. MMHC-LSHKDE Algorithm

MMHC-LSHKDE is an enhanced MMHC algorithm for continuous data BN structure learning. Its computational process is detailed in the Algorithm 2. MMHC-LSHKDE abandons the standard BIC scoring function, and we use LSHKDE-based conditional entropy to evaluate the quality of the network structure. In addition, instead of using the traditional chi-square test to measure the correlation between continuous variables, MMPC-LSHKDE applies LSHKDE to calculate the mutual information and conditional mutual information in Algorithm 3. These improvements make the strategy more adaptable to continuous data and improve its applicability.

The 15th procedure in Algorithm 2 uses add-edge, delete-edge, and reverse-edge to locally modify the current network structure *G*, obtaining a series of candidate network structures $G_{0}$. The algorithm only tries the operator add-edge: $x_{t}\to x$ if $x\in CPC(x_{t})$.
**Algorithm 2**: MMHC-LSHKDE algorithm**Input:** Dataset *D*; Variable set $X=\{x_{1},\ldots ,x_{n}\}$; Threshold value α
**Output:** DAG G=(X,E)

 1:
**Constraint phase:** 2: **for** 
$x_{t}\in X$ 
**do**
 3:    $CPC\left(x_{t}\right)=MMPC-LSHKDE\left(x_{t},D\right)$ using Algorithm 3
 4:    **for** $x\in \mathrm{CPC}(x_{t})$ **do** 5:           **if** $x_{t}\notin \mathrm{MMPC}-\mathrm{LSHKDE}\left(x,D\right)$ **then**
 6:           $CPC\left(x_{t}\right)=CPC\left(x_{t}\right)-\left\{x\right\}$ 7:        **else** 8:           $CPC\left(x_{t}\right)=CPC\left(x_{t}\right)$ 9:        **end if** 10:    **end for** 11: **end for** 12: **Search phase:** 13: Initialize network structure *G* 14: currentScore=Evaluate(G) using Equations (24)–(26) 15: $GenerateCandidates\left(G_{0}\right)=\left\{G_{1},G_{2},\ldots ,G_{n}\right\}$ 16: **for** 
$G_{i}\in G_{0}$ 
**do** 17:    $candidateScore=Evaluate(G_{i})$ using Equations (24)–(26) 18:    **if** candidateScore>currentScore **then** 19:        $G=G_{i}$ 20:        currentScore=candidateScore 21:        $G_{0}=GenerateCandidates\left(G\right)$ 22:    **else** 23:        break 24:    **end if** 25: **end for**


**Algorithm 3**: MMPC-LSHKDE algorithm**Input:** Dataset *D*; Variable set $X=\{x_{1},\ldots ,x_{n}\}$; Threshold value α **Output: **
$CPC(x_{t})$ 1: $CPC(x_{t})=\emptyset$ 2: **repeat** 3:    Calculation $AssocF=Max_{x\in X}Min_{CS\subseteq CPC(x_{t})}I\left(x,x_{t}|CS\right)$ using Equations (22) and (23) 4:    Calculation $x_{F}=\arg{Max}_{x\in X}Min_{CS\subseteq CPC(x_{t})}I\left(x,x_{t}|CS\right)$ using Equations (22) and (23) 5:    **if** AssocF≠0 **then** 6:        $\mathrm{CPC}(x_{t})=\mathrm{CPC}\left(x_{t}\right)\cup x_{F}$ 7:    **else** 8:        $CPC\left(x_{t}\right)=CPC\left(x_{t}\right)$ 9:    **end if** 10: **until** CPC has not changed 11: **for** 
$x\in CPC(x_{t})$ 
**do** 12:    **if** $\mathrm{CS}\subseteq \mathrm{CPC}(x_{t})$ and $I(x,x_{t}|CS)<\alpha$ **then** 13:        $CPC\left(x_{t}\right)=CPC\left(x_{t}\right)-\left\{x\right\}$ 14:    **else** 15:        $CPC\left(x_{t}\right)=CPC\left(x_{t}\right)$ 16:    **end if** 17: **end for**


## 5. Hybrid BNC Based on LSHKDE

In this section, we build the BNC and class the attributes as the parent nodes of all non-class attributes. By using the MMHC-LSHKDE algorithm, we analyze the dependency relationships between each non-class attribute to construct corresponding inference networks. Based on the input prior attribute variable values, we calculate the posterior probability of each category and select the category with the highest posterior probability as the classification result output [54].

Assuming the dataset contains *d* attributes, namely $x_{1},x_{2},\ldots ,x_{d}$ and a category attribute *C*, where the attribute can also be considered as a random variable, a BNC is to obtain the category corresponding to the maximum posterior probability [55].

(27) $$\hat{c}=\underset{c}{\arg\max}P\left(C|x_{1},x_{2},\ldots ,x_{d}\right)$$

By using the Bayesian theorem, we obtain the following equation:

(28) $$P\left(x_{1},x_{2},\ldots ,x_{d},C\right)=P\left(C\right)\prod_{i=1}^{n}P(x_{i}|\Pi \left(x_{i}\right))$$

where $\Pi (x_{i})$ represents the parent node of $x_{i}$.

We provide the calculation of conditional mutual information as shown in the following equations [56].

(29) $$\begin{array}{cc} I(x_{1},x_{2}|C) & =\;\int \int \;\sum_{c\in C}p\left(x_{1},x_{2},c\right)\log\frac{p(x_{1},x_{2}|c)}{p\left(x_{1}|c\right)p\left(x_{2}|c\right)}dx_{1}dx_{2}\\ \; & =\;\int \int \;\sum_{c\in C}p\left(x_{1},x_{2}\right)p\left(c|x_{1},x_{2}\right)\log\frac{p(x_{1},x_{2}|c)}{p\left(x_{1}|c\right)p\left(x_{2}|c\right)}dx_{1}dx_{2}\\ \; & =\frac{1}{n}\sum_{i=1}^{n}\sum_{c\in C}p\left(c|x_{i1},x_{i2}\right)\log\frac{p(x_{i1},x_{i2}|c)}{p\left(x_{i1}|c\right)p\left(x_{i2}|c\right)} \end{array}$$

(30) $$\begin{array}{cc} I(x_{1},x_{2}|C,x_{3}) & =\;\int \int \int \;\sum_{c\in C}p\left(x_{1},x_{2},x_{3},c\right)\log\frac{p(x_{1},x_{2}|c,x_{3})}{p\left(x_{1}|c,x_{3}\right)p\left(x_{2}|c,x_{3}\right)}dx_{1}dx_{2}dx_{3}\\ \; & =\;\int \int \int \;\sum_{c\in C}p\left(x_{1},x_{2},x_{3}\right)p\left(c|x_{1},x_{2},x_{3}\right)\log\frac{p(x_{1},x_{2}|c,x_{3})}{p\left(x_{1}|c,x_{3}\right)p\left(x_{2}|c,x_{3}\right)}dx_{1}dx_{2}dx_{3}\\ \; & =\frac{1}{n}\sum_{i=1}^{n}\sum_{c\in C}p\left(c|x_{i1},x_{i2},x_{i3},\right)\log\frac{p(x_{i1},x_{i2}|c,x_{i3})}{p\left(x_{i1}|c,x_{i3}\right)p\left(x_{i2}|c,x_{i3}\right)} \end{array}$$

The condition probability is shown in the following equation.

(31) $$P\left(x|c\right)=\frac{1}{L{\left(\sqrt{2\pi }h\right)}^{d}}\sum_{k=1}^{L}\frac{|bin_{ck}\left(x\right)|K\left(x,S_{ck}\right)}{L\mathrm{Pr}[hash\left(x\right)=hash\left(S_{ck}\right)]}$$

where $S_{ck}$ is the randomly selected sample in the bucket by using the *k* hash function for the category *C* dataset. The joint probability of KDE for event {C=c,X=x} occurring is shown in the following equation.   

(32) $$P\left(x,c\right)=P\left(c\right)P\left(x|c\right)=\frac{n_{c}}{n}\left(\frac{1}{L{\left(\sqrt{2\pi }h\right)}^{d}}\sum_{k=1}^{L}\frac{|bin_{ck}\left(x\right)|K\left(x,S_{ck}\right)}{L\mathrm{Pr}[hash\left(x\right)=hash\left(S_{ck}\right)]}\right)$$

The estimated probability of kernel density for the label {C=c} under the condition of {X=x} occurrence is shown in the following equation.

(33) $$P\left(c|x\right)=\frac{P(x,c)}{P(x)}=\frac{n_{c}\frac{1}{L{\left(\sqrt{2\pi }h\right)}^{d}}\sum_{k=1}^{L}\frac{|bin_{k}\left(x\right)|K\left(x,S_{ck}\right)}{L\mathrm{Pr}[hash\left(x\right)=hash\left(S_{ck}\right)]}}{n\frac{1}{L{\left(\sqrt{2\pi }h\right)}^{d}}\sum_{k=1}^{L}\frac{|bin_{k}\left(x\right)|K\left(x,S_{k}\right)}{L\mathrm{Pr}[hash\left(x\right)=hash\left(S_{k}\right)]}}=\frac{n_{c}\sum_{k=1}^{L}\frac{|bin_{ck}\left(x\right)|K\left(x,S_{ck}\right)}{\mathrm{Pr}[hash\left(x\right)=hash\left(S_{ck}\right)]}}{n\sum_{k=1}^{L}\frac{|bin_{k}\left(x\right)|K\left(x,S_{k}\right)}{\mathrm{Pr}[hash\left(x\right)=hash\left(S_{k}\right)]}}$$

where $S_{k}$ is the randomly selected sample in the bucket by using the *k* hash function for all samples. The classifier is shown in the Algorithm 4.
**Algorithm 4**: MMHC-LSHKDE-based BNC algorithm**Input:** Training dataset *D*, $X=node/C=\{x_{1},x_{2},\ldots ,x_{d}\}$ **Output:** BNC 1: Invoke the Algorithm 2 to perform network structure learning 2:      Calculate the mutual information in the Algorithm 2 using Equation (29) 3:      Calculate the conditional mutual information in the Algorithm 2 using Equation (30) 4: Add *C* as a parent node of each $x_{i}$ 5: Perform parameter learning and compute posterior probabilities for each category using Equation (28) 6: Select the category with the maximum posteriori probability as the classification result output


## 6. Experiment Results

### 6.1. Compare LSHKDE with KDE in Curve Fitting Performance

**Figure 2:**
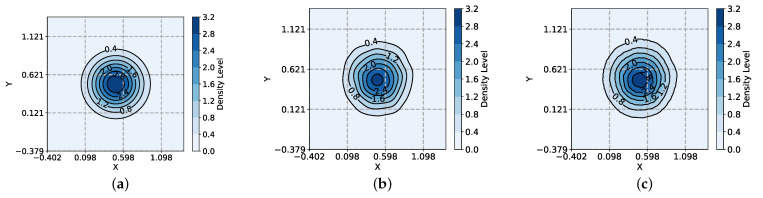
Estimation comparison of KDE and LSHKDE using a 2D dataset: (**a**) PDF. (**b**) KDE. (**c**) LSHKDE.

To verify the effectiveness of the LSHKDE Algorithm, we generates two-dimensional Gaussian samples. The mean value is $M=\left[\begin{array}{c} 0.5\\ 0.5 \end{array}\right]$ and the covariance is $S=\left[\begin{array}{cc} 0.05 & 0\\ 0 & 0.05 \end{array}\right]$. The sample number is 5000. All KDE use classic window widths [57]. The fitting results obtained from the above data are shown in Figure 2.

Figure 2 shows the contour plots of probability density function (PDF), KDE, and LSHKDE, where the contour lines represent different density levels, and the fill color corresponds to the probability density values. The color bar on the right further clarifies the mapping between colors and density values. As can be seen from the figure, it can be concluded that the density peak position fitted by LSHKDE is essentially the same as the position of the reference function. In terms of shape fitting, the estimation results of LSHKDE and KDE are very similar, with almost no difference observed under the condition of 5000 two-dimensional observer points.

We generated multidimensional data samples to compare the fitting accuracy and computational speed of traditional KDE and LSHKDE. By using the mean square error (MSE) metric, we evaluate the disparity between the fitted value and the reference density function. The smaller the value, the smaller the difference between the estimated value and the true value. It indicates a higher accuracy of the estimation. The specific parameters for generating data samples using different distributions are shown in Table 1. The data samples were fitted using LSH and LSHKDE and their corresponding MSE results are shown in Table 2.

According to Table 2, as the data dimension increases, the difference in fitting decreases. The fitting effect of LSHKDE on multidimensional data is similar to KDE, and its value gradually decreases. This paper selects simulated data generated by a d-dimensional standard Gaussian function to compare the fitting speed of KDE and LSHKDE.

**Figure 3:**
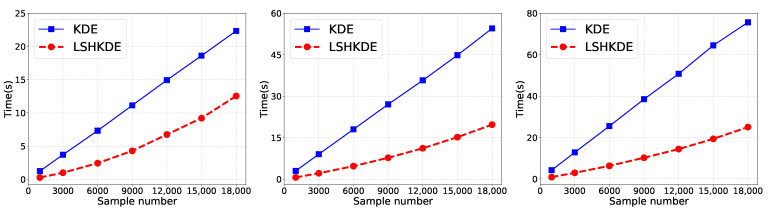
Comparison of time cost using different sample numbers.

As shown in Figure 3, as the sample size increases, our method has a lower computational time overhead than traditional KDE When the sample size reaches 18,000 points, our calculation time will not exceed 24 s compared to traditional KDE. Therefore, this experiment demonstrates that the LSH strategy can effectively reduce the required computational time overhead.

Compared to the traditional KDE, LSHKDE can perform KDE more efficiently by utilizing LSH techniques, significantly reducing computation time. In addition, LSHKDE has shown excellent performance in reducing storage space. By introducing a hash index storage structure, LSHKDE only needs to store information from the hash table, while regular KDE needs to maintain storage information for each data point. This storage method can effectively reduce the utilization of storage resources when processing large-scale data.

### 6.2. Comparison of BN Structure Learning Algorithms

#### 6.2.1. Datasets and Assessment Indicators

This paper primarily utilizes benchmark networks such as ALARM, CHILD, and ASIA to create datasets for testing and evaluating algorithm accuracy. Since the benchmark networks are used for data with discrete variables, we generate the continuous dataset required for the experiment through linear structural equations and the given network structure relationship [58].

(34) $$X=w_{x}^{T}Pa\left(X\right)+rand\left(0,1\right)$$

where the value of each variable *X* is a linear combination function of the value Pa(X) of its parent node and a random perturbation term. In this equation, the weight vector $w_{x}^{T}$ of variable *X* relative to the parent node is typically randomly generated, and the perturbation term is randomly generated from a uniform distribution between 0 and 1.

We measure the effectiveness of various algorithms by comparing the learned network structure with the reference network structure. Based on the obtained confusion matrix, corresponding relevant indicators can be calculated, including precision (*P*), recall (*R*), and the comprehensive indicator F1. Among them, accuracy measures the overall recognition accuracy, while recall reflects the proportion of edges correctly recognized by the model to all actual correct edges.

(35) $$P=\frac{TP}{TP+FP}\;R=\frac{TP}{TP+FN}\;F1=\frac{2PR}{P+R}$$

where TP is the number of correctly recognized edges, FP is the number of incorrectly recognized edges, and FN is the number of incorrectly recognized correct edges.

#### 6.2.2. Performance Comparison

**Figure 4:**
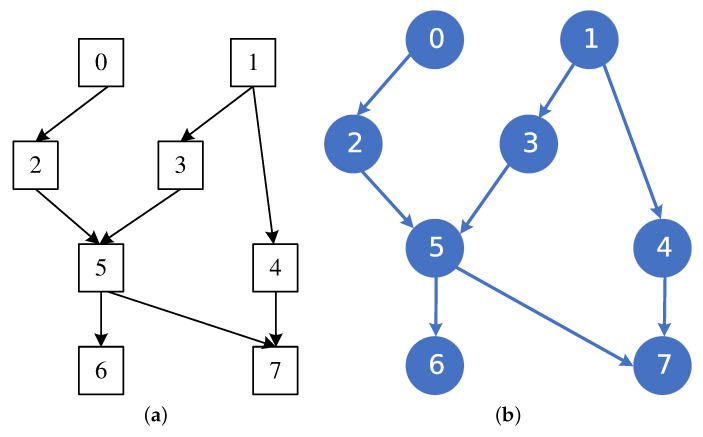
Comparison of ASIA network structure. (**a**) ASIA reference network. (**b**) ASIA learned network.

**Figure 5:**
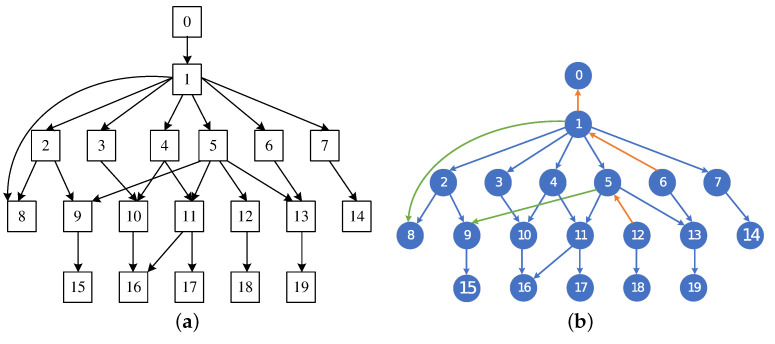
Comparison of CHILD network structure. (**a**) CHILD reference network. (**b**) CHILD learned network.

To evaluate the effectiveness of the algorithm proposed in this paper in the field of BN structure learning, we mainly selected three well-known network structures for our experiments. The AISA network is a small network and the network consists of eight nodes and eight edges. The CHILD network mainly consists of 20 nodes and 25 edges. The ALARM network mainly consists of 37 nodes and 46 edges. The first two networks are shown in Figure 4a and Figure 5a, and the network structures obtained by using the MMHC-LSHKDE algorithm are shown in Figure 4b and Figure 5b. In the figures, we highlight consistent edges in blue, mark reversed edges in orange-red, and represent missing edges in green.

From Figure 4 and Figure 5, the network design produced by the MMHC-LSHKDE algorithm largely corresponds to the conventional structure recognized in the field. It can accurately identify parent–child dependency relationships.

Meanwhile, we conducted a comprehensive comparison of the precision, recall, and F1 values of the MMHC algorithm, MMHC-KDE algorithm, and MMHC-LSHKDE algorithm on three recognized networks. The application of the traditional MMHC algorithm requires data discretization [59]. To ensure the accuracy of our experimental comparisons, we repeated the procedure 10 times. In each procedure, 5000 data samples were generated corresponding to each network. We then averaged their performance to serve as a baseline for analysis.

**Figure 6:**
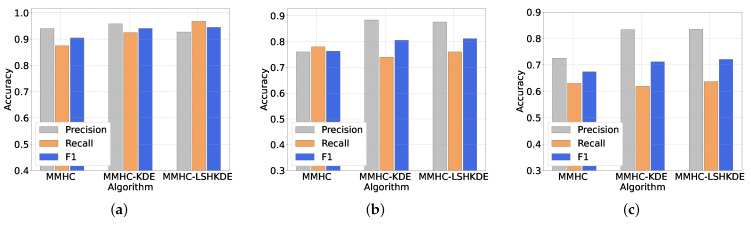
Comparison of algorithms under different datasets. (**a**) ASIA network dataset. (**b**) CHILD network dataset. (**c**) ALARM network dataset.

From Figure 6, we can observe a significant difference in the accuracy for the different networks. In the ASIA network, we specifically observed that the recall rates of MMHC-KDE and MMHC-LSHKDE algorithms are significantly superior to those of traditional MMHC algorithms. In the CHILD and ALARM networks, the precision of the MMHC-KDE and MMHC-LSHKDE algorithms is significantly higher than that of the MMHC algorithm. Overall, the MMHC-KDE and MMHC-LSHKDE algorithms perform similarly in accuracy. They are both superior to the MMHC algorithm. The main reason is the information loss caused by data discretization, which leads to a decrease in the accuracy of traditional MMHC algorithm.

**Figure 7:**
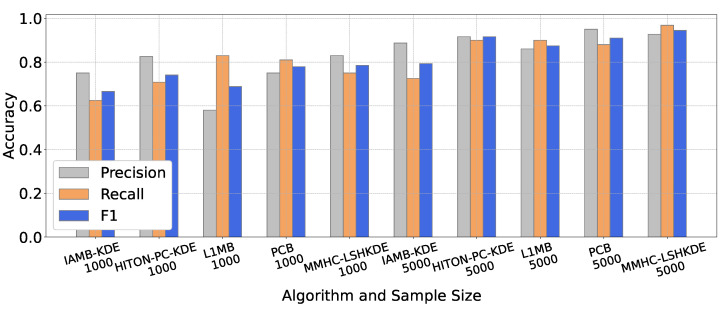
Accuracy comparison of different Bayesian network learning algorithms (ASIA).

**Figure 8:**
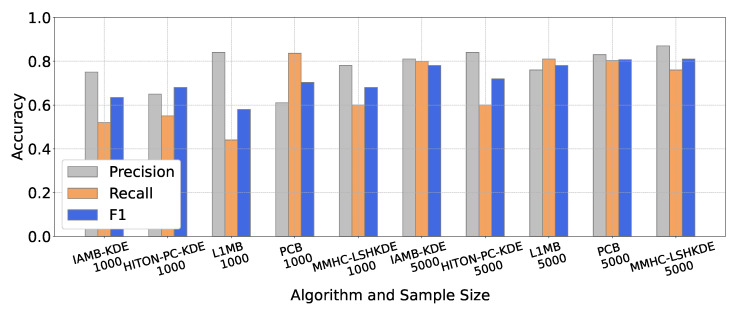
Accuracy comparison of different BN learning algorithms (CHILD).

**Figure 9:**
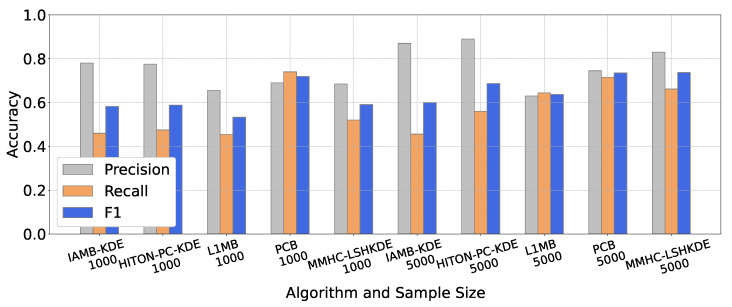
Accuracy comparison of different BN learning algorithms (ALARM).

To compare the learning performance of different algorithms, we selected five classic BN structure learning algorithms, which are IAMB-KDE [23], HITON-PC-KDE [24] (https://github.com/wt-hu/pyCausalFS), L1MB [19] (https://www.cs.ubc.ca/murphyk/, accessed on 20 January 2025), PCB [42], and our MMHC-LSHKDE, to construct the three benchmark networks mentioned above. The number of samples selected for each network is 1000 and 5000. From the results in Figure 7, Figure 8 and Figure 9, it is clear that there are significant differences in learning accuracy across different algorithms. Additionally, it can be observed that for a specific algorithm, the learning accuracy varies with the sample size. As the sample size increases, the learning accuracy of the five algorithms will generally be improved. Notably, for a sample size of 5000, the learning accuracy of the MMHC-LSHKDE algorithm is significantly higher than that of the other four algorithms.

**Figure 10:**
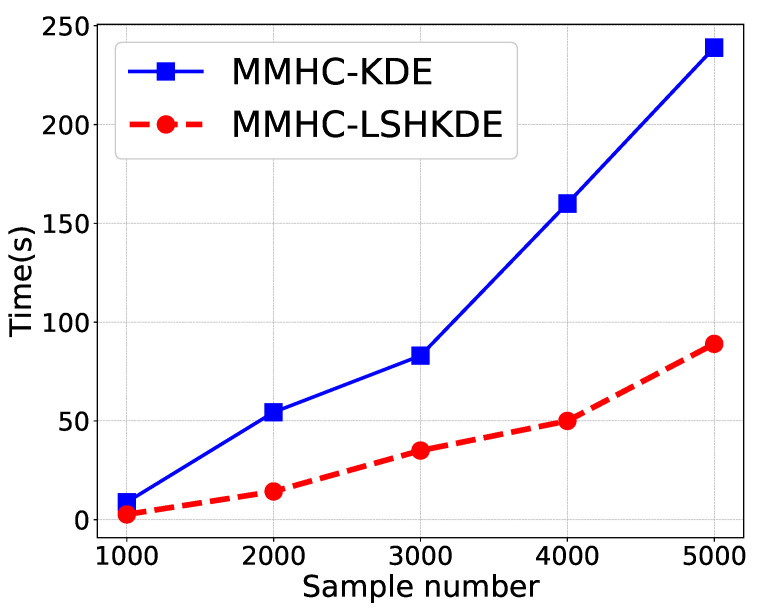
Learning time comparison using different MMHC structure learning algorithms.

Figure 10 shows the time cost comparison of the two algorithms. As the sample size increases, the learning time exhibits an upward trend. Compared with the MMHC-KDE algorithm, the execution time of the BN structure learning in MMHC-LSHKDE algorithm is significantly reduced. The temporal difference gradually expands with the increase in sample size. Especially when the sample size reaches 5000, the BN learning strategy of MMHC-LSHKDE algorithm shows a significant advantage over MMHC-KDE algorithm in terms of time efficiency. The advantage makes the MMHC-LSHKDE algorithm more suitable for handling large-scale datasets.

### 6.3. Classification Performance Comparison

The BNC, based on the BN structure learning algorithm, is widely used in the medical field. It not only requires a certain level of classification accuracy but also attracts attention due to its clear causal relationships and probabilistic interpretability. To validate the effectiveness of the LSHKDE-based BNC proposed in Section 5. We selects 20 consecutive classification datasets in the UCI data to complete classification comparison experiments. When encountering the issue of absent data points in the dataset, we implement a method of substituting them with the average value of each feature. The specific descriptive information of the dataset is shown in Table 3.

By selecting multiple classifiers to compare the classification performance, including naive Bayesian classifier (NBC), tree extended Bayesian classifier (TAN), flexible Bayesian classifier (FBC), k-nearest neighbor algorithm (KNN), decision tree C4.5, neural network (NN), support vector machine (SVM), 10 cross-validations is used in the experiment. The average classification accuracy is shown in Table 4.

From Table 4, we can observe that there are significant differences in the classification performance of different classifiers across the datasets. For most datasets, the performance of BNC outperforms that of several other classifiers. When the sample size is small, and reliable structure learning is not possible, classifiers such as the NBC and SVM are suitable. These classifiers rely on simple assumptions and are able to maintain a certain level of classification performance under limited data conditions. When the sample size reaches a certain threshold and the number of attribute features is large, the classification accuracy of the MMHC-LSHKDE-based BNC outperforms that of other classifiers. This classifier is able to more effectively capture the dependency structure between variables, thereby significantly improving classification performance. Overall, in terms of average classification accuracy, the MMHC-LSHKDE-based BNC outperforms the other seven classifiers with accuracy improvements of 5.8%, 2.3%, 4.3%, 3.6%, 5.5%, 1.6%, and 1%, respectively. Comparing the results with those of other classifiers, it is evident that the MMHC-LSHKDE-based BNC exhibits superior classification performance. Furthermore, it effectively addresses the challenges posed by increases in sample size and feature dimensions, especially with more complex datasets.

**Figure 11:**
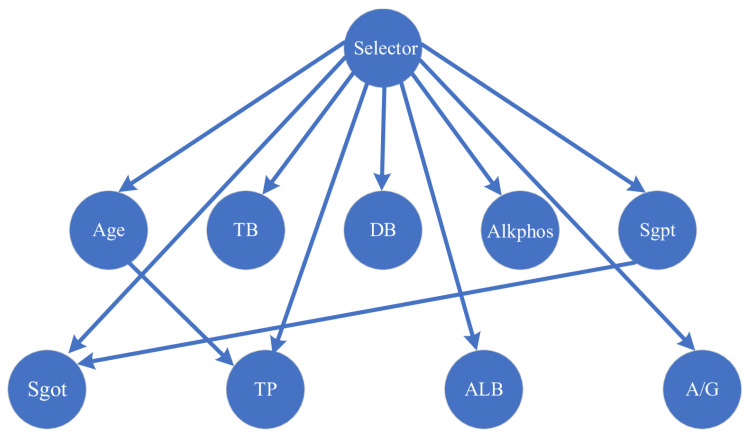
Indian liver patient dataset.

The MMHC-LSHKDE-based BNC has good interpretability, taking Indian liver disease as an example. The dataset of Indian liver disease contains records of 416 patients diagnosed with liver disease and 167 patients without liver disease. This information is contained in the class label named as "Selector". We used nine variables: age, total bilirubin (TB), direct bilirubin (DB), total protein (TP), albumin (ALB), albumin-to-globulin ratio (A/G), alamine aminotransferase (Sgpt), aspartate aminotransferase (Sgot), and alkaline phosphatase (Alkphos). From Figure 11, it is evident that various biochemical indicators related to liver disease, such as age, are correlated when determining the presence of liver disease. When liver cells are damaged, enzymes are released in large quantities into the bloodstream, causing an increase in the indicators of alkaline phosphatase, alanine aminotransferase, and aspartate aminotransferase. When liver cells undergo degeneration and necrosis, and bilirubin metabolism is disrupted, the levels of total bilirubin and direct bilirubin will increase. Albumin, total protein, and the ratio of albumin to globulin can reflect the synthesis function of the liver and can be used to detect chronic liver injury. However, we can also observe a dependency relationship between alanine aminotransferase and aspartate aminotransferase, irrespective of the occurrence of liver disease. When alanine aminotransferase increases, aspartate aminotransferase also increases. There is a dependency relationship between age and albumin. As age increases, the albumin content in the body also increases. Based on the graph, we can discover the causal relationship between those features.

## 7. Conclusions

This paper utilizes KDE-based mutual information and conditional mutual information to calculate the correlations between different variables, enabling the skeleton learning of BN structures without assuming data distribution. By using the heuristic algorithm, conditional entropy is used as a scoring function for searching the optimal network structure. We provide a new computational method for speeding up the computation using LSH functions, thereby reducing the time required for BN structure learning. The effectiveness of our method is demonstrated by three experimental tests. Without decreasing estimation accuracy, LSHKDE achieves a significant improvement in computational speed compared to traditional KDE. The MMHC-LSHKDE algorithm can achieve higher accuracy in learning BN structures compared to the traditional algorithm. The MMHC-LSHKDE-based BNC demonstrates high accuracy and strong interpretability compared to other classifiers. Subsequent research is necessary to choose the optimal number of hash functions *L* to improve the efficiency of BN structure learning. Future research will focus on improving algorithm parameter optimization methods to meet the needs of diverse datasets.

## Figures and Tables

**Table 1:** Parameter settings for different distributions.

Distribution	Gaussian	T-Distribution	Cauchy	Laplace
d dimension	μ=[$\mu_{1}$, …, $\mu_{d}$]; $\mu_{1}$=$\mu_{d}$=0; δ= $\left[\begin{array}{ccc} \delta_{1} & \cdots & 0\\ \vdots & \ddots & \vdots \\ 0 & \cdots & \delta_{d} \end{array}\right]$; $\delta_{1}$= $\delta_{d}$=1;	$loc=[l_{1},\ldots ,l_{d}]$; $l_{1}=l_{d}=0$; $scale=\left[\begin{array}{ccc} s_{1} & \cdots & 0\\ \vdots & \ddots & \vdots \\ 0 & \cdots & s_{d} \end{array}\right]$; df=5; $s_{1}=s_{d}=1$	$loc=[l_{1},\ldots ,l_{d}]$; $l_{1}=l_{d}=0$; $scale=\left[\begin{array}{ccc} s_{1} & \cdots & 0\\ \vdots & \ddots & \vdots \\ 0 & \cdots & s_{d} \end{array}\right]$; df=1; $s_{1}=s_{d}=1$	$loc=[l_{1},\ldots ,l_{d}]$; $l_{1}=l_{d}=0$; Σ = $\left[\begin{array}{ccc} b_{1} & \cdots & 0\\ \vdots & \ddots & \vdots \\ 0 & \cdots & b_{d} \end{array}\right]$; $b_{1}$=$b_{d}=2$

**Table 2:** Estimation comparsion using different distribution data.

Distribution Function	Model	d = 10	d = 30	d = 50
Gaussian Distribution	KDE	2.12 × 10^−7^	3.21 × 10^−18^	1.03 × 10^−28^
	LSHKDE	1.98 × 10^−7^	3.20 × 10^−18^	1.03 × 10^−28^
T-distribution	KDE	1.27 × 10^−6^	5.55 × 10^−14^	1.56 × 10^−20^
	LSHKDE	1.24 × 10^−6^	5.55 × 10^−14^	1.56 × 10^−20^
Cauchy distribution	KDE	4.55 × 10^−6^	1.00 × 10^−14^	4.22 × 10^−32^
	LSHKDE	4.54 × 10^−6^	1.00 × 10^−14^	4.21 × 10^−32^
Laplace distribution	KDE	1.19 × 10^−7^	6.18 × 10^−19^	2.41 × 10^−31^
	LSHKDE	1.09 × 10^−7^	6.18 × 10^−19^	2.40 × 10^−31^

**Table 3:** Description of the datasets used in experiments.

NO.	Dataset	Instances	Attributes	Class
1	Abalone	4177	8	3
2	Cmc	1473	6	3
3	Ecoli	292	5	4
4	Fires	244	11	2
5	Glass	214	9	6
6	Haberman	306	3	2
7	Ilpd	583	9	2
8	Ionosphere	351	34	2
9	Iris	150	4	3
10	Maternal	1013	6	3
11	Parkinsons	195	22	2
12	Pima	768	8	2
13	Raisin	900	7	2
14	Red wine	1599	11	5
15	Transfusion	748	4	2
16	Wdbc	569	30	2
17	Wholesale	440	6	3
18	Wine	178	13	3
19	Wpbc	198	33	2
20	Yeast	1484	6	4

**Table 4:** Comparison of classification accuracy of different classifiers.

Dataset Name	NBC	TAN	FBC	KNN	C4.5	NN	SVM	BNC
Abalone	0.518 ± 0.066	0.498 ± 0.056	0.502 ± 0.064	0.520 ± 0.062	0.488 ±0.055	0.516 ±0.065	0.543 ± 0.069	**0.644**± 0.068
Cmc	0.470 ± 0.029	0.491 ± 0.032	0.485 ± 0.023	0.484 ± 0.029	0.475 ±0.044	0.508 ± 0.038	0.511 ± 0.036	**0.517**± 0.032
Ecoli	0.910 ± 0.040	0.900 ± 0.036	0.905 ± 0.042	**0.913**± 0.040	0.862 ± 0.049	0.909 ± 0.032	0.875 ± 0.034	0.875 ± 0.041
Fire	0.942 ± 0.044	0.916 ± 0.042	0.902 ± 0.042	0.922 ± 0.034	**0.976**± 0.028	0.934 ± 0.046	0.946 ±0.038	0.920 ± 0.036
Glass	0.636 ± 0.116	0.698 ± 0.096	0.474 ± 0.099	0.520 ± 0.105	0.641 ± 0.112	0.547 ± 0.095	0.690 ± 0.121	**0.717**± 0.093
Haberman	0.745 ± 0.079	0.755 ± 0.072	0.745 ± 0.065	0.722 ± 0.083	0.644 ±0.077	0.755 ± 0.086	0.735 ± 0.090	**0.767**± 0.082
Ilpd	0.554 ± 0.086	0.678 ± 0.079	0.648 ± 0.072	0.663 ± 0.084	0.649 ± 0.083	0.703 ± 0.067	0.715 ±0.062	**0.844**± 0.070
Ionosphere	0.751 ± 0.079	0.712 ± 0.066	0.682 ± 0.056	0.740 ± 0.062	0.761 ± 0.061	0.765 ± 0.075	**0.781**± 0.065	0.705 ± 0.068
Iris	0.946 ± 0.061	0.926 ± 0.067	0.951 ± 0.068	0.933 ± 0.063	0.941 ± 0.073	0.931 ± 0.062	**0.960**± 0.064	0.920 ± 0.065
Maternal	0.583 ± 0.076	0.663 ± 0.088	0.623 ± 0.092	0.682 ± 0.072	0.674 ± 0.095	0.587 ± 0.083	0.591 ± 0.090	**0.686**± 0.075
Parkinsons	0.669 ± 0.056	**0.875**± 0.054	0.798 ± 0.060	0.772 ± 0.041	0.778 ± 0.064	0.797 ± 0.062	0.833 ± 0.049	0.754 ± 0.059
Pima	0.755 ± 0.054	0.765 ± 0.043	0.755 ± 0.043	0.744 ± 0.056	0.718 ± 0.049	0.766 ± 0.050	0.768 ± 0.054	**0.792**± 0.049
Raisin	0.827 ± 0.029	0.835 ± 0.023	0.805 ± 0.020	0.830 ± 0.039	0.793± 0.035	0.865 ± 0.026	0.833 ± 0.025	**0.867**± 0.024
Red wine	0.541 ± 0.046	0.530 ± 0.045	0.560 ± 0.052	0.526 ± 0.062	0.487 ± 0.048	0.587 ± 0.059	0.575 ± 0.064	**0.588**± 0.050
Transfusion	0.751 ± 0.152	**0.784**± 0.142	0.760 ± 0.112	0.763 ± 0.140	0.731 ± 0.138	0.774 ± 0.121	0.762 ± 0.155	0.768 ± 0.132
Wdbc	0.926 ± 0.039	0.962 ± 0.017	0.934 ± 0.021	0.963 ± 0.025	0.940 ± 0.030	0.970 ± 0.011	**0.977**± 0.016	0.946 ± 0.020
Wholesale	0.500 ± 0.197	0.718 ± 0.156	0.715 ± 0.201	0.609 ± 0.122	0.534 ± 0.110	0.715 ± 0.103	0.718 ± 0.099	**0.718**± 0.102
Wine	**0.964**± 0.029	0.880 ± 0.023	0.943 ± 0.037	0.938 ± 0.044	0.904 ±0.045	0.961 ±0.027	0.944 ±0.038	0.905 ± 0.039
Wpbc	0.681 ± 0.079	0.645 ± 0.073	0.750 ± 0.068	0.747 ± 0.075	0.655 ± 0.068	0.758 ± 0.072	0.759 ± 0.070	**0.764**± 0.069
Yeast	0.512 ± 0.048	0.582 ± 0.050	0.497 ± 0.039	0.576 ± 0.053	0.531 ± 0.046	**0.589**± 0.052	0.588 ± 0.045	0.568 ± 0.042
Average	0.706 ± 0.070	0.741 ± 0.063	0.721 ±0.063	0.728 ± 0.064	0.709 ± 0.065	0.748 ± 0.061	0.754 ± 0.064	**0.764**± 0.060

## Data Availability

The data that support the findings of this study are available in UCI dataset https://archive.ics.uci.edu/datasets and Kaggle dataset https://www.kaggle.com/datasets. The code of the paper is in the following link https://github.com/liu1328/MMHC-LSHKDE (accessed on 10 January 2025).
